# Risk factors for infection at the saphenous vein harvest site after coronary artery bypass grafting surgery: a retrospective cohort study

**DOI:** 10.1186/s13019-024-02799-4

**Published:** 2024-05-31

**Authors:** Hanna Unosson, Maria Hälleberg Nyman, Karin Falk Brynhildsen, Örjan Friberg

**Affiliations:** 1 Department of Cardiothoracic and Vascular Surgery, Faculty of Medicine and Health, Örebro University, Örebro, Sweden; 2 Faculty of Medicine and Health, School of Health Sciences, Örebro University, Örebro, Sweden; 3 Faculty of Medicine and Health, University Health Care Research Centre, Örebro University, Örebro, Sweden; 4 Department of Cardiothoracic and Vascular Surgery, Örebro University Hospital, Örebro, Sweden

**Keywords:** Female sex, Leg wound infection, Smoking

## Abstract

**Introduction:**

Surgical site infection after saphenous vein harvest is common, with reported leg wound infection rates ranging from 2 to 24%. There have been few investigations into sex-related differences in complication rates. Moreover, varied effects of smoking have been reported. The aim of this study was to investigate risk factors such as gender and smoking, associated with surgical site infection after vein graft harvesting in coronary artery bypass grafting surgery.

**Methods:**

We included 2,188 consecutive patients who underwent coronary artery bypass grafting surgery with at least one vein graft at our centre from 2009 to 2018. All patients were followed up postoperatively. Risk factors for leg wound infection requiring antibiotic treatment and surgical revision were analysed using logistic regression analysis.

**Results:**

In total, 374 patients (17.1%) received antibiotic treatment and 154 (7.0%) underwent surgical revision for leg wound infection at the harvest site. Female sex, high body mass index, diabetes mellitus, longer operation time, peripheral vascular disease and direct oral anticoagulants were independently associated with any leg wound infection at the harvest site. Among surgically revised patients, female sex and insulin or oral treatment for diabetes mellitus as well as longer operation time were independent risk factors. Smoking was not associated with leg wound infection.

**Conclusion:**

Female sex is associated with increased risk of leg wound infection. The underlying mechanism is unknown. In the current population, previous or current smoking was not associated with an increased risk of leg wound infection.

## Background

In coronary artery bypass grafting (CABG) surgery the saphenous vein is the most common conduit for coronary revascularization, being used in 80% of cases [1]. Surgical site infection (SSI) at the saphenous vein harvest site is more common than sternal wound infections, with reported leg wound infection (LWI) rates ranging from 2 to 24% [2–5]. Leg wound infections have a negative effect on patients’ morbidity, quality of life and wellbeing [6]; they prolong hospitalization, may require extensive debridement and surgical procedures and are an economic burden to the health care system [6, 7]. To reduce the risk of LWI, measures such as antibiotic prophylaxis, proper skin disinfection and maintaining an aseptic environment are applied [7–9]. Risk factors associated with LWI include external factors such as the vein harvesting technique [10–12], duration of surgery [13], number of vein grafts [14] and choice of sutures [15–17]. Patient-dependent risk factors for LWI include diabetes mellitus (DM), obesity, peripheral vascular disease, impaired renal function and low left ventricular ejection fraction (LVEF) [13, 14, 18].

In different surgical procedures, men are often more affected by SSI than women [19]. Studies regarding sex as a risk factor for sternal wound infection after cardiac surgery show divergent results [4, 5, 20]. However, it has been reported that leg wound complications are actually more common in female patients [14, 18, 21], although further studies are warranted to confirm this.

Smoking is a modifiable risk factor (that is, a behavioral risk factor) that has been associated with wound complications after different types of surgery [22, 23]. Studies investigating LWI after CABG surgery are sparse and no association between smoking and LWI has yet been shown [3, 14].

In conclusion, ambiguity persists regarding the understanding of some potential risk factors for LWI. This study aimed to investigate risk factors for LWI after vein graft harvesting in CABG, with focus on female sex and smoking.

**Table 1:** Baseline characteristics of, and operative factors in, coronary artery bypass grafting (CABG) patients with or without leg wound infection (LWI) at the saphenous vein harvesting site

	Total	No LWI	Any LWI	Surgical revision
	*n* = 2,188	*n* = 1,814 (82.9%)	*n* = 374 (17.1%)	*n =* 154 (7.0%)
**Patient factors**				
**Sex, n (%)**				
Female	439 (20)	335 (18.5)	104 (27.8)	48 (31.2)
Male	1,749 (80)	1,479 (81.5)	270 (72.2)	106 (68.8)
**Age, yrs, n (%)**				
0–50	65 (3.0)	52 (2.9)	13 (3.5)	4 (2.6)
51–60	305 (13.9)	256 (14.1)	49 (13.1)	16 (10.4)
61–70	821 (37.5)	693 (38.2)	128 (34.2)	67 (43.5)
71–80	843 (38.5)	685 (37.8)	158 (42.2)	56 (36.4)
81–100	154 (7.1)	128 (7.0)	26 (7.0)	11 (7.1)
**Body mass index, kg/m**^**2**^, **n (%)**				
<18.5	6 (0.3)	3 (0.1)	3 (0.8)	0 (0.0)
18.5–<25	461 (21.1)	409 (22.6)	52 (14.0)	19 (12.3)
25–<30	1,052 (48.1)	893 (49.3)	159 (42.5)	77 (50.0)
≥30	668 (30.5)	508 (28.0)	160 (42.7)	58 (37.7)
Missing	1 (0.0)	1 (0.0)	0 (0.0)	0 (0.0)
**Diabetes mellitus, n (%)**				
No	1,488 (68.0)	1,281 (70.6)	207 (55.3)	72 (46.8)
Insulin-treated	352 (16.1)	248 (13.7)	104 (27.8)	51 (33.1)
Tablet-treated	276 (12.6)	223 (12.3)	53 (14.2)	29 (18.8)
Diet-treated	63 (2.9)	54 (3.0)	9 (2.4)	2 (1.3)
Missing	9 (0.4)	8 (0.4)	1 (0.3)	0 (0.0)
**Haemoglobin, g/L, n (%)**				
≤120	278 (12.7)	217 (12.0)	61 (16.3)	31 (20.2)
≥121	1,902 (87.0)	1,590 (87.6)	312 (83.4)	122 (79.2)
Missing	8 (0.3)	7 (0.4)	1 (0.3)	1 (0.6)
**Renal function, (n) %**				
Normal function	929 (42.5)	776 (42.8)	153 (41.0)	60 (38.9)
Mild reduction	885 (40.5)	733 (40.4)	152 (40.6)	63 (41.0)
Moderate reduction	337 (15.4)	272 (15.0)	65 (17.4)	28 (18.2)
Severe reduction	25 (1.1)	23 (1.3)	2 (0.5)	2 (1.3)
Renal failure	11 (0.5)	9 (0.5)	2 (0.5)	1 (0.6)
Missing	1 (0.0)	1 (0.0)	0 (0.0)	0 (0.0)
**Smoking, n (%)**				
Smoker	232 (10.6)	184 (10.1)	48 (12.8)	25 (16.2)
Ex-smoker^a^	1,016 (46.4)	847 (46.7)	169 (45.2)	62 (40.3)
Never smoker	878 (40.1)	732 (40.4)	146 (39.0)	59 (38.3)
Missing	62 (2.9)	51 (2.8)	11 (3.0)	8 (5.2)
**LVEF, n (%)**				
>50%	1,596 (73.0)	1,342 (74.0)	254 (68.0)	101 (65.6)
41–50%	324 (14.8)	262 (14.4)	62 (16.6)	26 (16.9)
31–40%	162 (7.4)	128 (7.1)	34 (9.1)	17 (11.0)
≤30%	95 (4.3)	72 (4.0)	23 (6.1)	10 (6.5)
Missing	11 (0.5)	10 (0.5)	1 (0.2)	0 (0.0)
**Anticoagulants, n (%)**				
Heparin/LMWH	482 (22.0)	394 (21.7)	88 (23.5)	31 (20.3)
DOAC	81 (3.7)	60 (3.3)	21 (5.6)	9 (5.8)
No	1,602 (73.2)	1,343 (74.0)	259 (69.3)	110 (71.4)
Missing	23 (1.1)	17 (1.0)	6 (1.6)	4 (2.5)
**Peripheral vascular disease, n (%)**				
Yes	149 (6.8)	113 (6.3)	36 (9.6)	17 (11.0)
No	2,028 (92.7)	1,693 (93.3)	335 (89.6)	137 (89.0)
Missing	11 (0.5)	8 (0.4)	3 (0.8)	0 (0.0)
**Operative factors**				
**Urgency, n (%)**				
Emergency (within 24 h)	97 (4.4)	79 (4.4)	18 (4.8)	10 (6.5)
Urgent	779 (35.6)	638 (35.2)	141 (37.7)	57 (37.0)
Elective	1,292 (59.0)	1,082 (59.6)	210 (56.2)	86 (55.9)
Missing	20 (1.0)	15 (0.8)	5 (1.3)	1 (0.6)
**Type of intervention**, **n (%)**				
CABG	1,724 (78.8)	1,441 (79.5)	283 (75.7)	121 (78.6)
CABG + valve	408 (18.6)	331 (18.2)	77 (20.6)	26 (16.9)
CABG + other surgery	56 (2.6)	42 (2.3)	14 (3.7)	7 (4.5)
**Vein harvesting technique, n (%)**				
No-touch technique	1,633 (74.6)	1,345 (74.1)	288 (77.0)	119 (77.3)
Conventional technique	555 (25.4)	469 (25.9)	86 (23.0)	35 (22.7)
**Number of graft vessels, n (%)**				
1–2	946 (43.2)	804 (44.3)	142 (38.0)	59 (38.3)
3–4	1,167 (53.4)	956 (52.7)	211 (56.4)	82 (53.3)
5–6	75 (3.4)	54 (3.0)	21 (5.6)	13 (8.4)
**Operation time, n (%)**				
<3 h	400 (18.3)	353 (19.5)	47 (12.6)	17 (11.0)
3–5 h	1,444 (66.0)	1,200 (66.2)	244 (65.2)	92 (59.7)
>5 h	323 (14.8)	242 (13.3)	81 (21.7)	43 (28.0)
Missing	21 (0.9)	19 (1.0)	2 (0.5)	2 (1.3)

BMI = body mass index; CABG = coronary artery bypass grafting; DOAC **=** direct oral anticoagulant; LMWH = low-molecular-weight heparin; LVEF = left ventricular ejection fraction

Peripheral vascular disease = one or more of: claudication, carotid occlusion or 50% stenosis, previous or planned intervention on the abdominal aorta, limb arteries or carotids, or amputation for arterial disease

^a^Ex-smoker > 1 month

## Materials and methods

### Study design

This was a retrospective cohort study based on prospectively collected data from a local quality register at a single cardiothoracic centre in Sweden.

### Data sources

The Carath Registry is a quality register including pre-, intra- and postoperative patient data. Our study included the following variables: sex, age, body mass index (BMI), glomerular filtration rate (GFR), preoperative haemoglobin (HB), smoking status, DM, vein harvesting technique (conventional or “no-touch” technique, where the vein is harvested along with the perivascular adipose tissue) [24], number of distal anastomoses, LVEF, peripheral vascular disease, degree of urgency (elective or acute), preoperative anticoagulant treatment, type of surgery (CABG or CABG + valve) and operation time. These 15 variables, identified from the Carath Registry, were evaluated regarding their association with LWI. They were selected based on previous research and the authors’ own clinical experience, and to ensure inclusion of some less investigated factors.

**Table 2:** Baseline characteristics of and operative factors in the included women and men

	Women, *n* = 439	Men, *n* = 1,749	*P*-value
**Patient factors**			
**Age, yrs, median (min–max)**	70.0 (37–89)	69.0 (30–87)	0.078
**BMI, kg/m**^**2**^, **n (%)**^a^			0.305
<18.5	2 (0.5)	4 (0.2)	
18.5–<25	106 (24.1)	355 (20.3)	
25–<30	168 (38.3)	884 (50.6)	
≥30	163 (37.1)	505 (28.9)	
**Diabetes mellitus, n (%)** ^a, f^			0.002
Yes	165 (37.8)	526 (30.2)	
No	272 (62.2)	1,216 (69.8)	
**Haemoglobin, g/L, n (%)** ^b, g^			<0.001
≤120	137 (31.4)	141 (8.0)	
≥121	299 (68.6)	1,603 (92.0)	
**Renal function, n (%)** ^d^			<0.001
Normal function	123 (28.0)	806 (46.1)	
Mild reduction	190 (43.3)	695 (39.8)	
Moderate reduction	110 (25.1)	227 (13.0)	
Severe reduction	11 (2.5)	14 (0.8)	
Renal failure	5 (1.1)	6 (0.3)	
**Smoking, n (%)** ^c, h^			<0.001
Smoker	64 (15.2)	168 (9.8)	
Ex-smoker^1^	160 (38.1)	856 (50.2)	
Never smoker	196 (46.7)	682 (40.0)	
**LVEF, n (%)** ^d, i^			0.828
>50%	315 (72.0)	1,281 (73.7)	
41–50%	74 (16.9)	250 (14.4)	
31–40%	30 (6.8)	132 (7.6)	
≤30%	19 (4.3)	76 (4.3)	
**Anticoagulants, n (%)** ^b, j^			0.073
Heparin/LMWH	113 (25.9)	369 (21.3)	
DOAC	12 (2.8)	69 (4.0)	
No	311 (71.3)	1,291 (74.7)	
**Peripheral vascular disease, n (%)** ^a, k^			0.817
Yes	31 (7.1)	118 (6.8)	
No	406 (92.9)	1,622 (93.2)	
**Operative factors**			
**Urgency, n (%)** ^e, l^			0.002
Emergency (within 24 h)	27 (6.2)	70 (4.0)	
Urgent	179 (41.2)	600 (34.6)	
Elective	229 (52.6)	1,063 (61.4)	
**Type of intervention, n (%)**			0.006
CABG	324 (73.8)	1,400 (80.1)	
CABG + valve	105 (23.9)	303 (17.3)	
CABG + other surgery	10 (2.3)	46 (2.6)	
**Vein harvesting technique, n (%)**			<0.001
No-touch technique	298 (67.9)	1,335 (76.3)	
Conventional technique	141 (32.1)	414 (23.7)	
**Number of graft vessels, n (%)**			0.052
1–2	211 (48.1)	735 (42.0)	
3–4	212 (48.3)	955 (54.6)	
5–6	16 (3.6)	59 (3.4)	
**Operation time, n (%)** ^b, m^			0.286
<3 h	78 (17.9)	322 (18.6)	
3–5 h	284 (65.1)	1,160 (67.0)	
>5 h	74 (17.0)	249 (14.4)	

BMI = body mass index; CABG = coronary artery bypass grafting; DOAC = direct oral anticoagulant; LMWH = low-molecular-weight heparin; LVEF = left ventricular ejection fraction

Peripheral vascular disease = one or more of: claudication, carotid occlusion or 50% stenosis, previous or planned intervention on the abdominal aorta, limb arteries or carotids, or amputation for arterial disease

One character behind a variable indicates missing data in the men’s group; two characters indicate missing data in both groups: ^a^Missing data on two patients, ^b^missing data on three patients, ^c^missing data on 19 patients, ^d^missing data on one patient, ^e^ missing data on four patients, ^f^missing data on seven patients, ^g^missing data on five patients, ^h^missing data on 43 patients, ^I^missing data on ten patients, ^j^missing data on 20 patients, ^k^missing data on nine patients, ^l^missing data on 16 patients, ^m^missing data on 18 patients

^1^Ex-smoker >1 month

### Patients and perioperative routines

Follow-up data were obtained regarding consecutive patients undergoing CABG alone or CABG in combination with other cardiac surgical procedures, with at least one vein graft, between 1 January 2009 and 31 December 2018 at the Department of Vascular and Cardiothoracic Surgery, Örebro University Hospital, Örebro, Sweden [25].

Preoperatively all elective and acute patients showered with chlorhexidine gluconate (4%). Hair cutting was performed with a hair clipper at the department ward the day before surgery or directly before the operation. Vein harvesting was performed using an open surgical technique. The wound was closed with two or three layers of subcutaneous and intracutaneous monofilament sutures. Between 2009 and 2013, Biosyn 3 − 0® (Covidien, Minneapolis, MN, USA) was used; from 2014, triclosan-coated Monocryl 3 − 0® monofilament sutures (Ethicon; Johnson & Johnson, Cincinnati, OH, USA) were used. All patients were treated with perioperative intravenous antibiotic prophylaxis. During 2009–2014, patients received cloxacillin 2 g, starting 25 min before the sternal skin incision. Two hours after the initial dose, another dose of 2 g was administered. For lengthy operations, the dose was repeated every 6 h. Before sternal closure, a final intraoperative dose was given if the previous dose had been administered ≥ 2 h previously. Postoperatively, 2 g of cloxacillin was administered every 8 h for a total duration of at least 24 h. From 2015 onward, a 3 g single dose of benzyl penicillin, administered together with the preoperative dose of cloxacillin, was added. Clindamycin 600 mg was given preoperatively to patients allergic to beta-lactam antibiotics and the dose was repeated after 4 h of surgery, with further postoperative doses given every 8 h for a total of 24 h.

**Table 3:** Results of the univariable and multivariable logistic regression analysis of risk factors for any leg wound infection (LWI)

	Unadjusted analysis			Adjusted analysis^#^		
	OR	95% CI	*P*-value	OR	95% CI	*P*-value
**Characteristics**						
**Sex**						
Female	1.70	1.31–2.19	<0.001	1.62	1.22–2.16	<0.001
Male	Ref					
**Age, yrs**						
0–50	1.08	0.57–2.03	0.803			
51–60	0.83	0.58–1.17	0.298			
61–70	0.80	0.62–1.03	0.089			
71–80	Ref					
81–100	0.88	0.55–1.38	0.585			
**BMI, kg/m** ^**2**^						
<18.5	7.86	1.54–39.98	0.013	4.48	0.81–24.68	0.085
18.5–<25	Ref					
25–<30	1.40	1.00–1.95	0.048	1.35	0.95–1.91	0.089
≥30	2.47	1.76–3.47	<0.001	2.04	1.42–2.93	<0.001
**Diabetes mellitus**						
No	Ref					
Insulin-treated	2.59	1.97–3.40	<0.001	2.13	1.58–2.85	<0.001
Tablet-treated	1.47	1.05–2.05	0.023	1.26	0.88–1.80	0.203
Diet-treated	1.03	0.50–2.12	0.933	0.90	0.42–1.96	0.808
**Haemoglobin, g/L**						
≤120	1.43	1.05–1.95	0.023	1.10	0.77–1.55	0.592
≥121	Ref					
**Renal function**						
Normal function	Ref					
Mild reduction	1.05	0.82–1.34	0.688			
Moderate reduction	1.21	0.87–1.67	0.241			
Severe reduction	0.44	0.10–1.89	0.270			
Renal failure	1.12	0.24–5.26	0.879			
**Smoking**						
Never smoker	Ref					
Ex-smoker ^a^	1.00	0.78–1.27	0.998	0.97	0.75–1.25	0.825
Smoker	1.30	0.90–1.88	0.148	1.12	0.76–1.66	0.554
**LVEF**						
>50%	Ref					
41–50%	1.25	0.91–1.70	0.155			
31–40%	1.40	0.94–2.09	0.098			
≤30%	1.68	1.03–2.75	0.036			
**Anticoagulants**						
Heparin/LMWH	1.15	0.88–1.51	0.280	1.15	0.86–1.52	0.331
DOAC	1.81	1.08–3.03	0.023	1.98	1.13–3.45	0.015
No	Ref					
**Peripheral vascular disease, n (%)**						
No	Ref					
Yes	1.61	1.08–2.38	0.018	1.59	1.05–2.42	0.028
**Operative factors**						
**Degree of urgency**						
Emergency (within 24 h)	1.17	0.68-2.00	0.555			
Urgent	1.13	0.90–1.44	0.278			
Elective	Ref					
**Type of intervention**						
CABG	Ref					
CABG + valve	1.18	0.89–1.56	0.234			
CABG + other surgery	1.69	0.91–3.14	0.093			
**Vein harvesting technique**						
No-touch technique	1.16	0.89–1.51	0.247			
Conventional technique	Ref					
**Number of anastomoses**						
1–2	0.80	0.63–1.01	0.060	0.79	0.61–1.01	0.064
3–4	Ref					
5–6	1.75	1.04–2.98	0.035	1.24	0.68–2.26	0.466
**Operation time**						
<3 h	0.65	0.46–0.91	0.013	0.76	0.54–1.08	0.138
3–5 h	Ref					
>5 h	1.64	1.23–2.19	<0.001	1.36	1.00–1.86	0.050

BMI = body mass index; CABG = coronary artery bypass grafting; CI = confidence interval; DOAC = direct oral anticoagulant; LMWH = low-molecular-weight heparin; LVEF = left ventricular ejection fraction; OR = odds ratio

Peripheral vascular disease = one or more of: claudication, carotid occlusion or 50% stenosis, previous or planned intervention on the abdominal aorta, limb arteries or carotids, or amputation for arterial disease

^a^Ex-smoker > 1 month

^#^The model is adjusted for sex, diabetes mellitus, BMI, haemoglobin, smoking, anticoagulants, peripheral vascular disease, number of anastomoses and operation time

### Definition and follow-up of surgical site infection

The study’s two outcome variables were all LWIs, and surgical revision for LWI. In this study, LWI was defined as any wound complication for which the patient had been prescribed antibiotic treatment, in accordance with the Centers for Disease Control and Prevention (CDC) classification where one single criterion for superficial surgical site infection is “diagnosis of a superficial incisional SSI by a physician or physician designee” [26]. An LWI that had been surgically revised (under local or general anaesthesia) was considered a more severe wound infection; these LWIs were analysed separately. All surgically revised LWIs also required antibiotic treatment.

**Table 4:** Multivariable logistic regression analysis of risk factors for leg wound infection (LWI) requiring surgical revision

	Unadjusted analysis			Adjusted analysis^#^		
	OR	95% CI	*P*-value	OR	95% CI	*P*-value
**Characteristics**						
**Sex** Female Male Ref	1.90	1.33–2.72	<0.001	1.74	1.16–2.61	0.007
**Age, yrs**						
0–50	0.92	0.32–2.62	0.878			
51–60	0.77	0.43–1.37	0.390			
61–70	1.24	0.86–1.80	0.238			
71–80	Ref					
81–100	1.08	0.55–2.11	0.820			
**Diabetes mellitus**						
No Insulin-treated	Ref 3.33	2.28–4.87	<0.001	2.95	1.95–4.46	<0.001
Tablet-treated	2.30	1.47–3.62	<0.001	2.26	1.40–3.65	<0.001
Diet-treated	0.64	0.15–2.69	0.547	0.69	0.16–2.94	0.625
**BMI, kg/m** ^**2**^						
BMI <18.5	NE					
BMI 18.5–<25	Ref					
BMI 25–<30	1.83	1.09–3.07	0.020	1.83	1.06–3.17	0.028
BMI ≥ 30	2.21	1.29–3.76	0.003	1.68	0.95–2.98	0.074
**Haemoglobin, g/L**						
≤120	1.83	1.20–2.77	0.004	1.29	0.81–2.06	0.282
≥121	Ref					
**Renal function**						
Normal function	Ref					
Mild reduction	1.11	0.77–1.60	0.576			
Moderate reduction	1.31	0.82–2.09	0.254			
Severe reduction	1.25	0.29–5.46	0.758			
Renal failure	1.44	0.18–11.5	0.726			
**Smoking**						
Ex-smoker^a^	0.90	0.62–1.30	0.584	0.88	0.60–1.29	0.526
Smoker	1.67	1.02–2.74	0.040	1.33	0.78–2.25	0.291
Never smoker	Ref					
**LVEF**						
>50%	Ref					
41–50%	1.29	0.82–2.02	0.264			
31–40%	1.73	1.01–2.98	0.046			
≤30%	1.74	0.87–3.45	0.113			
**Anticoagulants**						
Heparin/LMWH	0.93	0.61–1.4	0.739			
DOAC	1.69	0.82–3.48	0.150			
No	Ref					
**Peripheral vascular disease**						
Yes	1.77	1.04–3.03	0.035	1.66	0.94–2.93	0.080
No	Ref					
**Operative factors**						
**Degree of urgency**						
Emergency (within 24 h)	1.61	0.80–3.21	0.175			
Urgent	1.10	0.78–1.56	0.566			
Elective	Ref					
**Type of intervention**						
CABG	Ref					
CABG + valve	0.90	0.58–1.39	0.643			
CABG + other surgery	1.89	0.83–4.26	0.124			
**Vein harvesting technique**						
No-touch technique	1.16	0.79–1.72	0.436			
Conventional technique	Ref					
**Number of anastomoses**						
1–2	0.88	0.62–1.24	0.470	0.96	0.66–1.38	0.832
3–4	Ref					
5–6	2.77	1.46–5.25	0.002	1.63	0.77–3.48	0.199
**Operation time**						
Operation time <3 h	0.65	0.38–1.10	0.114	0.69	0.39–1.20	0.197
Operation time 3–5 h						
Operation time >5 h	2.25	1.53–3.31	<0.001	1.75	1.15–2.67	0.009

BMI = body mass index; CABG = coronary artery bypass grafting; CI = confidence interval; DOAC = direct oral anticoagulant; LMWH = low-molecular-weight heparin; LVEF = left ventricular ejection fraction; OR = odds ratio; NE = not estimatable

Peripheral vascular disease = one or more of: claudication, carotid occlusion or 50% stenosis, previous or planned intervention on the abdominal aorta, limb arteries or carotids, or amputation for arterial disease

^a^Ex-smoker > 1 month

^#^The model is adjusted for sex, diabetes mellitus, BMI, haemoglobin, smoking, peripheral vascular disease, number of anastomoses and operation time

A registered nurse made a postoperative follow-up call 2 months after the surgery to check for any postoperative infections. A simplified questionnaire based on the validated Additional treatment, Serous discharge, Erythema, Purulent exudate, Separation of deep tissues, Isolation of bacteria, and Stay as inpatient prolonged over 14 days (ASEPSIS) score [27] was used to classify patient-reported postoperative symptoms of infection.

### Statistical analysis

Categorical variables were expressed as numbers and percentages, and age as median and range. All categorical variables were compared using chi-squared test; for age, Mann-Whitney U test was used. Before the analyses, all ratio data were categorized. Age was divided into decades. Univariable and multivariable logistic regression analyses were used to analyse the relationship of the predictor variables with the outcome. Predictors with p-values ≤ 0.2 in the univariable analysis were added to the multivariable model. The results from the multivariable analysis were reported as adjusted odds ratios (ORs) with 95% confidence intervals (CIs). A p-value of < 0.05 was considered statistically significant.

The statistical analyses were performed using IBM® SPSS® Statistics version 27 (IBM Corp., Armonk, NY, USA).

### Ethics

The study was approved by the Swedish Ethical Review Authority (ID 2020–03103). Because of the retrospective nature of the study no informed consent was obtained.

## Results

In total, 2,628 patients underwent CABG surgery during the study period. Of these, 2,188 patients with at least one vein graft were followed up. The study population consisted of 439 women (20%) and 1,749 men (80%), their ages ranging from 30 to 89 (median 70) years. Approximately 60% of the group were smokers or ex-smokers and the majority had a BMI ≥ 25. Altogether 374 (17.1%) patients received antibiotic treatment for any LWI; 154 (7.0%) also underwent surgical revision under local or general anaesthesia. Baseline characteristics are presented in Table 1.

The incidence of LWI at the harvest site was 23.7% in women and 15.4% in men (Table 1). Diabetes mellitus and anaemia were also more common among the women, as was impaired GFR. Moreover, there were more active smokers in the female group. The majority of the surgeries were elective but there was a tendency for the women to have more urgent operations as well as more combined interventions (CABG + valve surgery). Further differences between the sexes are described in Table 2.

### Risk factors for any leg wound infection

In the multivariable logistic regression analysis, female sex [p<0.001; OR 1.62 (95% CI 1.22–2.16)], BMI ≥ 30 [p<0.001; OR 2.04 (95% CI 1.42–2.93)], insulin-treated DM [p<0.001; OR 2.13 (95% CI 1.58–2.85)], peripheral vascular disease [*p* = 0.028; OR 1.59 (95% CI 1.05–2.42)] and direct oral anticoagulants (DOACs) [*p* = 0.015; OR 1.98 (95% CI 1.13–3.45)] were independently associated with any LWI (Table 3). Smoking was not associated with any statistically significant increased risk for LWI when added to the multivariable risk factor analysis. For additional details, see Table 3.

### Risk factors for leg wound infection requiring surgical revision

In the multivariable analysis, female sex [*p* = 0.007; OR 1.74 (95% CI 1.16–2.61)], insulin-treated DM [p<0.001; OR 2.95 (95% CI 1.95–4.46)], tablet-treated DM [p<0.001; OR 2.26 (95% CI 1.40–3.65)] and operation time >5 h [*p* = 0.009; OR 1.75 (95% CI 1.15–2.67)] were independent risk factors for surgical revision. For further details, see Table 4.

## Discussion

To date, only a few studies have investigated risk factors for LWI rate after CABG surgery [14]. We found female sex, medically treated DM, high BMI, DOAC treatment, and duration of surgery, as well as presence of peripheral vascular disease, to be independent predictive risk factors for LWI. The finding that female sex was an independent risk factor for LWI confirms earlier studies [14, 18, 21]. The underlying causes are unknown, but various assumptions and theories have been put forward as to why women have a higher incidence of LWI and other complications in vascular and cardiothoracic surgery. In other surgical procedures, men are more affected by SSI [19]. One theory, based on our own experiences, is that women in general have thinner skin on the legs than men, and therefore the skin is more fragile and difficult to heal. However, there are conflicting opinions about sex differences regarding the thickness of the epidermis. A recently published review article demonstrates weak evidence for this sex difference hypothesis [28]. Earlier research described the complexity of wound healing, pointing out that there is not one single explanation for all disparities [19, 29]. Wound healing is a complex physiological process. Disturbances in this process can lead to worse or delayed wound healing. Oestrogen deficiency has been shown to be detrimental for the wound healing process and postmenopausal women have an increased risk of complications as a result [30]. One hypothesis could be that the delayed wound healing process allows more time for bacteria to contaminate the wound. Oestrogen treatment could possibly reverse these effects of delayed wound healing [30]. Further, there is a theory that the different fat distribution in women and men affects both wound healing after surgery [31] and the outcome of the surgical procedure. Impaired peripheral circulation in women compared with men may be another explanation for women’s increased risk of LWI [32].

In our study, smoking was not found to be a significant risk factor for LWI. Previous research on smoking and its impact on SSIs after different types of surgery has shown conflicting results [3, 22, 23]. A large study including data on major general surgical procedures indicated that smoking increases the risk of postoperative SSI and wound disruption [22]. Regardless of sex, age, duration of surgery or anaesthetic technique, current smoking was associated with a higher risk of postoperative wound complications compared with not smoking [22]. By contrast, and in line with the results of our study, smoking has not been shown to be an independent risk factor in previous studies on LWI after CABG surgery [14, 33]. Despite this, one study pointed out that leg wound healing disturbances in the form of wound edge necrosis and dehiscence were significantly higher in smokers than in non-smokers; but there were no differences between these two groups for LWIs [3]. Based on the findings of our study, in addition to earlier research, smoking can hardly be considered an important risk factor for occurrence of LWI after CABG.

## Limitations

Our study was a single-centre study, which could potentially be a limitation regarding the external validity. An earlier study including national data from all cardiothoracic surgery centres in Sweden indicated that local traditions may be among the most important factors determining which procedures are employed in the operating theatre [34]. Such traditions would likely affect the external factors (operation time, type of sutures, vein harvesting technique, suturing experience, etc.) in the vein harvesting procedure. Furthermore, there are several issues with the definition of “smokers” and “ex-smokers”, in terms of number of cigarettes per day and also of the smoke-free period for individuals to be termed “ex-smokers”. We did not have data on the smoke-free period. In other words, our ex-smokers could have stopped at any point between 1 month and 15 years previously.

The greatest strengths of this study are the high number of included patients and the quality of the registry.

## Conclusion

In this study, female sex was an independent predictor for SSI at the harvest site after CABG surgery. The underlying mechanisms need to be further investigated. Previous or current smoking was not indicated as a risk factor for occurrence of LWI.

## Data Availability

The datasets used and/or analyzed during the current study are available from the corresponding author on reasonable request.
